# Giant Zero-Drift Electronic Behaviors in Methylammonium Lead Halide Perovskite Diodes by Doping Iodine Ions

**DOI:** 10.3390/ma11091606

**Published:** 2018-09-04

**Authors:** Tiqiang Pang, Renxu Jia, Yucheng Wang, Kai Sun, Ziyang Hu, Yuejin Zhu, Suzhen Luan, Yuming Zhang

**Affiliations:** 1 School of Microelectronics, Xidian University, Key Laboratory of Wide Band-Gap Semiconductor Materials and Devices, Xi’an 710071, China; tqpang@stu.xidian.edu.cn (T.P.); ycwang_23@stu.xidian.edu.cn (Y.W.); luansuzhen@126.com (S.L.); zhangym@xidian.edu.cn (Y.Z.); 2 Department of Microelectronic Science and Engineering, Ningbo University, Ningbo 315211, China; sky1250565776@sina.com (K.S.); zhuyuejin@nbu.edu.cn (Y.Z.)

**Keywords:** Methylammonium lead halide perovskite, iodine doped, zero-point drift

## Abstract

Methylammonium lead halide perovskites have attracted extensive attention for optoelectronic applications. Carrier transport in perovskites is obscured by vacancy-mediated ion migration, resulting in anomalous electronic behavior and deteriorated reliability of the devices. In this communication, we demonstrate that ion migration can be significantly enhanced by doping additional mobile I^-^ ions into the perovskite bulk. Ionic confinement structures of vertical metal oxide semiconductor (MOS) and lateral metal semiconductor metal (MSM) diodes designed to decouple ion-migration/accumulation and electronic transport are fabricated and characterized. Measurement conditions (electric-field history, scan rate and sweep frequency) are shown to affect the electronic transport in perovskite films, through a mechanism involving ion migration and accumulation at the block interfaces. Prominent zero-point drifts of dark current-voltage curves in both vertical and lateral diode are presented, and further varied with the perovskite film containingthe different iodine-lead atomic ratio. The doped perovskite has a large ion current at grain boundaries, offering a large ion hysteresis loopand zero drift value. The results confirmthat the intrinsic behavior of perovskite film is responsible for the hysteresisof the optoelectronic devices, but also paves the way for potential applications in many types of devices including memristors and solid electrolyte batteries by doping the native species (I^−^ ions) in perovskite film.

## 1. Introduction

In the past decade, solar cells (PSCs) based on hybrid organic-inorganic perovskites and other optoelectronic devices have made great progress [1,2,3,4].These achievements are attributed to the distinguished optoelectronic properties of perovskites, such as high photoelectric conversion efficiencies, low radiation, high charge carrier mobility, high gain, and mixed ionic-electronic conductivity [2,5,6,7,8,9,10]. However, there is still a lack of comprehensive understanding of anomalous optoelectronic behaviors, such as the so-called giant dielectric constant [11], current-voltage (*I-V*) hysteresis [12], and field-/light-induced phase segregation [6,13]. Although there is currently no universally accepted mechanism that can explain this phenomenon unanimously, the research done so far has indeed provided a deeper understanding of the subject. It is accepted by most researchers that current hysteresis may be an intrinsic property of perovskite materials that may originate from the electromigration of ions because of its low activation energy, which has been verified by experimental and theoretical simulations [6,9,14,15,16,17,18,19]. In addition, the diffusion of intrinsic ion defects in organometal halide perovskites has an important influence on the performance of perovskite devices [9,12,17,20,21,22,23].

A lot of effort has been made to suppress current hysteresis and improve solar cell efficiency. The modification of the interface between the perovskite and the electron collecting layer obviously affects the hysteresis in a device made of perovskite [24,25,26,27,28]. Shao et al. [8] found that a fullerene layer deposited on the perovskite can effectively passivate charge trap states and eliminate the notorious current hysteresis. Xu et al. [29] found that Phenyl-C_61_-butyric acidmethyl ester (PCBM) is evenly distributed in the perovskite grain boundary and can passivate the pivotal PbI_3_^−^ antisite defects in the perovskite self-assembly process. In our previous report, doping PCBM in perovskite would suppress zero drift, which could increase the efficiency of detectors [30]. The position of zero possesses a large drift under dark conditions, and it is a typical phenomenon observed in mixed ionic-electronic conductors that the voltage is not 0 V at the minimum current.

However, thinking in the opposite direction, increasing the zero drift may make it more suitable for manufacturing resistive switching devices [23,30,31,32,33,34,35]. Iodine ions are considered to be the culprit of dark current hysteresis due to their low activation energy (0.1–0.6 eV) [14,16,17] and diffusion energy barrier (~0.17 eV) [21].Here, we intentionally change the iodine content in the perovskite by additive native species (iodine ions); an iodine-doped perovskite was introduced [36,37]. It should be noted that normal photovoltaic devices with nonlinear *I-V* processes complicate the electron-ion hybrid transport. To address this issue, we adopted a simplified capacitor-like architecture that combines the ionic movement between two opposite block layers. A metal oxide semiconductor (MOS) vertical diode with Al/Si/SiO_2_/perovskite/Au and a metal semiconductor metal (MSM) lateral diode with Au as the two metal electrodes were fabricated. All the measurements were performed under dark conditions to isolate the ionic-electronic transport in perovskite without their being obscured by photogenerated carriers and complicated device structures with electron- and hole-transport layers. The migration and accumulation of these ions led to modification of the interfacial charge properties, which are reflected in the field-direction and scan-rate dependency of the *I-V* measurements. Prominent zero-point drifts in both the vertical and lateral diode structures are presented, and these varied further with the iodine-lead atomic ratio in perovskite. The iodine-doped perovskite has a large ion current at the grain boundaries, which is responsible for the large ion hysteresis (currents) and zero drift value in the iodine-doped perovskite-based diodes.

## 2. Materials and Methods

### 2.1. Preparation of the Iodine-Doped Perovskite and Pristine Perovskite

The iodine-doped perovskite film and pristine perovskite used in this study were deposited using a one-step solution method. First, methylammonium iodine (MAI) and lead iodine (PbI_2_) precursors are dissolved in *N*, *N*-dimethylformamide (DMF) which contains DMSO (PbI_2_:MAI:DMSO = 1:1:4) with the molar ratio 1:1 to form the perovskite precursor solution. The precursor solution of perovskite was prepared with DMF containing iodine (Pb:I = 1:1% molecule). Next, the precursor solutions were spin-coated onto the well-treated substrate at a rate of 4000 rpm for 30 s in a nitrogen glove box. Then, the substrates were heated at 100 °C for 4 h to crystallize. After cooling, the as-prepared films were washed with isopropanol and dried at 65 °C for 5min. Finally, the wet-mixed solutions were annealed at 80 °C for 40 min. To obtain a clean and uniform interface, the surfaces of substrates were successively ultrasonically cleaned with acetone and ethanol and deionizedwater for 3 min, respectively, and dried with dry nitrogen, followed by treatment with ultraviolet ozone (UV-O_3_). SiO_2_ surface layers (300 nm) were obtained by thermal oxidation, with n-type heavy doped Si (500 μm) wafers being used as the substrates in the experiments of electrical characteristics, XPS and c-AFM. Fluorine-doped tin oxide (FTO) substrates were used in the experiments of SEM, XRD, UV-visible, PL, and TRPL.

### 2.2. Characterization of Perovskite Films

A scanning electron microscope (JSM-6700F, JEOL, Tokyo, Japan) was used to acquire surface SEM images of the CH_3_NH_3_PbI_3_ films, where a collection energy of 5 kV and magnification of 10,000 times were applied.The X-ray diffraction patterns of the crystallized CH_3_NH_3_PbI_3_ perovskite films were reported using an XRD-7000 diffractometer (Shimadzu Corp, Tokyo, Japan) with Cu Kα target radiation with a power of 2.7 kW. The scanning angle for the measurements were in the range 10°–50°; the step scan mode was used, and the scanning speed was set as 8°/min. The absorption spectra were used for testing the absorption of the perovskite films grown on a FTO substrate using an ultraviolet-visible spectrometer (Lambda40p, PerkinElmer, Waltham, MA, USA) with a wavelength range from 450 to 850 nm. Steady-state fluorescence spectroscopy was used to studied the fluorescence and transient properties of carriers of perovskite films (FLS980, Edinburgh Instruments Company, London, UK). The XPS depth profile (KratosAxis UltraDLD spectrometer, Kratos Analytical Ltd, Manchester, UK) was obtained using a single alternating 68 s sputtering with 1 keV Ar^+^ beam and XPS analysis using 400 μm spot size monochromatic Al-K alpha radiation. The quantification obtained after the Shirley background was performed using the Avantage software to subtract the pass energy over the measurement spectrum obtained at 200 eV with anenergy step size of 1 eV. Conductive atomic force microscopy(c-AFM) (Asylum Research MFP-3D Large Sample AFMs, Oxford, Asylum Research, UK) was carried out in an ultrahigh-vacuum using Cr/Pt-coated silicon cantilevers (Multi75E-G, Budget Sensor, Sofia, Bulgaria). All the measurements were performed at a background pressure of <2 × 10^−10^ Torr after transferring the samples from ambient conditions without any additional treatment. The current-voltage measurements of the devices were performed using a Keysight B1500A semiconductor parameter analyzer (Keysight Technologies, Calabasas, CA, USA) and a Signotone Micromanipulator S-1160 probe station (Bridge Technology, Chandler Heights AZ, USA) under dark conditions with electrostatic shielding. During the measurements, the devices were kept at room temperature in the ambient atmosphere. 

## 3. Results and Discussion

**Figure 1:**
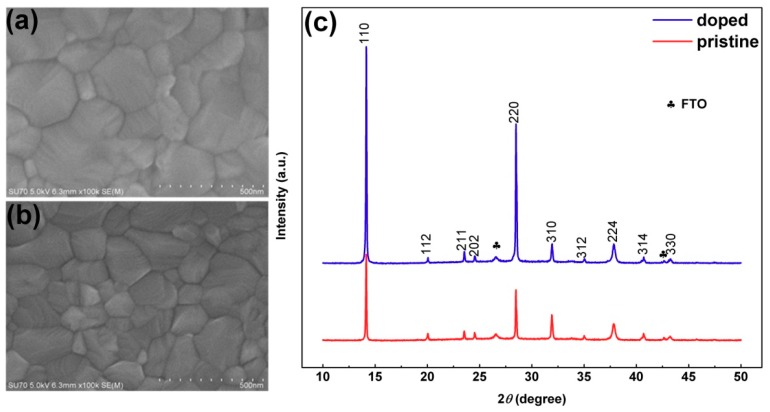
Comparison of material characterization between iodine-doped perovskites and pristine perovskites. (**a**) and (**b**) are the top SEM of iodine-doped perovskite and pristine perovskite, respectively; (**c**) X-ray diffraction pattern of the pristine perovskite film (the red line) and the iodine-doped perovskite film (the blue line) grown on FTO substrate.

The CH_3_NH_3_PbI_3_ perovskite films were fabricated by a one-step spin-coating process, followed by 40 min of thermal annealing, as shown in the dotted box of Figure S1; the doping procedure is shown in the solid box. For adetailed description of the preparation process, see the experimental section. To understand the effect of iodine doping on the growth and crystallization of perovskite films, the surface topography and the crystalline characteristics were studied by scanning electron microscopy (SEM) and X-ray diffraction (XRD) patterns. Figure 1a,b are the top SEM images of iodine-doped perovskite and pristine perovskite, respectively. The grain size of the iodine-doped perovskite film is significantly better than that of the pristine perovskite film, which indicates that the additional I^-^ promotes the formation of the perovskite film. These two films cover the substrate fully and uniformly, avoiding the risk of short circuits in the measurement. Figure 1c shows the XRD of the pristine perovskite film (the red line) and the iodine-doped perovskite film (the blue line) grown on fluorine-doped tin oxide(FTO) substrate; it can be found that the iodine-doped perovskite crystallizes in a tetragonal crystal structure, and its diffraction peaks are consistent with those of the pristine perovskite film, but the crystallinity is higher than the latter, which confirmed that the crystallization of perovskite is strongly affected by the iodine ion content [35]. UV-visible absorption spectrum, steady state and transient fluorescence spectroscopy were employed to investigate the optical properties (Figures S2–S4); for the details, see the supporting materials. 

**Figure 2:**
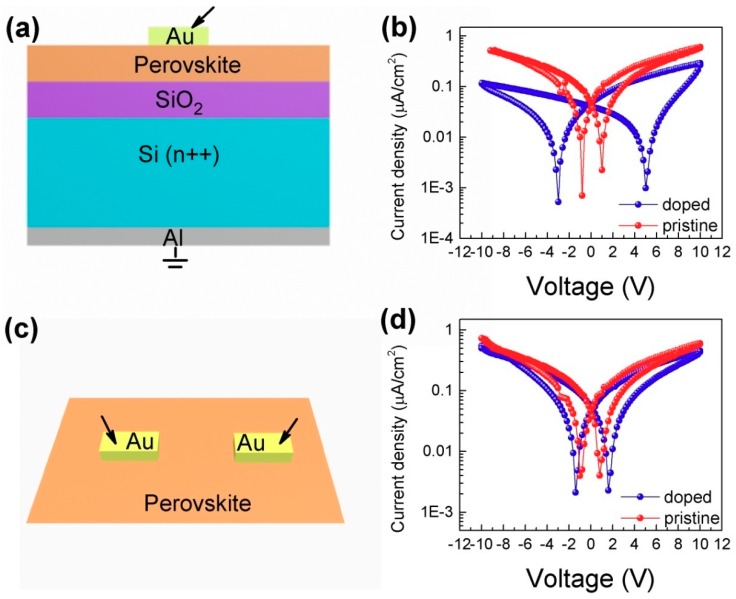
*I-V* characteristics of iodine-doped perovskite and pristine perovskite based on different device structures in the dark. (**a**) Al/Si/SiO_2_/perovskite/Au vertical structure. (**b**) *I-V* hysteresis curves of iodine-doped perovskite and pristine perovskite based on the Al/Si/SiO_2_/perovskite/Au vertical structure. (**c**) Au/perovskite/Au lateral structure. (**d**) *I-V* hysteresis curves of iodine-doped perovskite and pristine perovskite based on the Au/perovskite/Au lateral structure.

**Figure 3:**
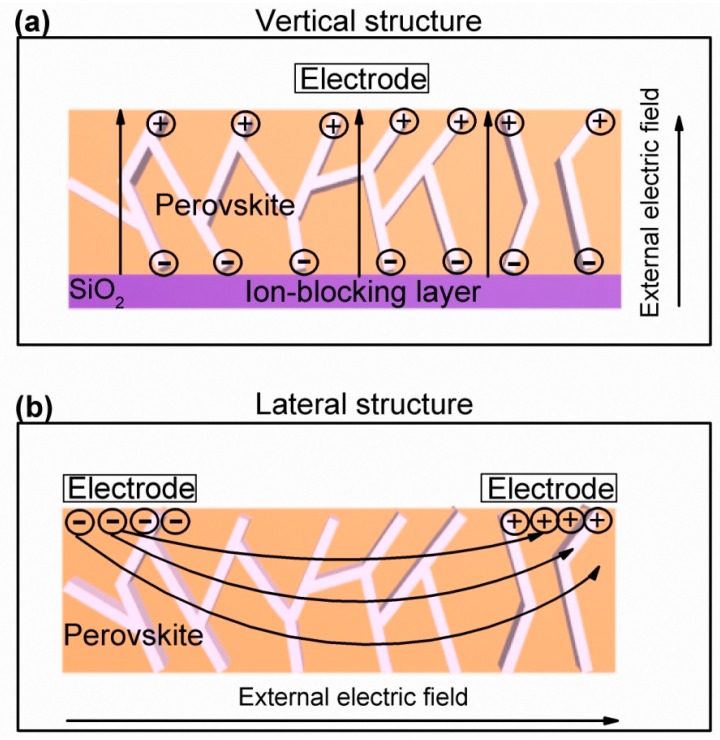
The schematic diagram of ion migration paths: (**a**) vertical structure; (**b**) lateral structure.

Due to the migration of defect ions by external electric fields, perovskites exhibit hysteresis in their *I-V* characteristics [9,14,15,17,21]. To study the doped properties of the perovskite film with native species under the influence of external electric fields, *I-V* measurements were performed. Figure 2b shows representative sets of the dark *I-V* curves of iodine-doped perovskite and pristine perovskite based on the Al/Si/SiO_2_/perovskite/Au vertical structure, as shown in Figure 2a. A typical device consists of ~300 nm thick perovskite films deposited on a SiO_2_/Si substrate with prefabricated Al (200 nm) bottom electrodes, followed by the deposition of the Au top electrodes (~160 nm thick) using the magnetron sputtering method with shallow masks. That is, the ionic conductive channel of this structure is located in the bulk of the perovskites. The voltage drift of iodine-doped perovskite-based devices (>4 V) is significantly greater than that of pristine perovskite-based devices (<1 V) at the same sweep rate for the same structure. From the linear coordinates (Figure S5), it can be seen that the iodine-doped perovskite-based device has a larger hysteretic loop than that of pristine perovskite. Device structure has a great influence on the photovoltaic switching mechanism because of the preferred orientation of perovskite materials [15]. To elaborate the movement paths of ions, the MSM structure that conductive channel on the perovskite surface was fabricated as shown in Figure 2c. The device incorporates ~300 nm thick perovskite films deposited on a SiO_2_/Si substrate with the deposition of the Au block electrodes (~160 nm) thick with the interval of 200 μm. Unlike the MOS diode, the positive and negative drift voltages of the MSM diode are almost identical due to the symmetry of the device structure, as shown in Figure 2d. The zero drift of the iodine-doped perovskite devices (~2 V) is still larger than that of the pristine perovskite devices (~1 V). The conducting channel in the bulk of perovskite suggests that zero drift is only related to perovskite materials. More importantly, regardless of the perovskite, the zero drift of the conductive path on the surface is less than that of the conductive path in the bulk of the perovskite, independent of doping. In the vertical structure, the electric field is basically parallel to the grain boundary, and the ions are more severely accumulated at the grain boundary, resulting in alarge zero drift. However, in the lateral structure, the direction of the electric field is perpendicular to the grain boundary, and the ions are also accumulated, but not very seriously, so the zero drift is not large. The corresponding schematic diagramsare shown in Figure 3a,b. In short, the ion movement dominates in the vertical structure; that is, the perovskite bulk is the main path for ion transport. For the MSM diodes, the magnitude of the measured current at positive and negative voltages is almost the same, indicating no apparent rectification in these ohmic contacted diodes. This implies that no built-in field or shifted energy alignment occurred in heterojunctions under dark conditions.

**Figure 4:**
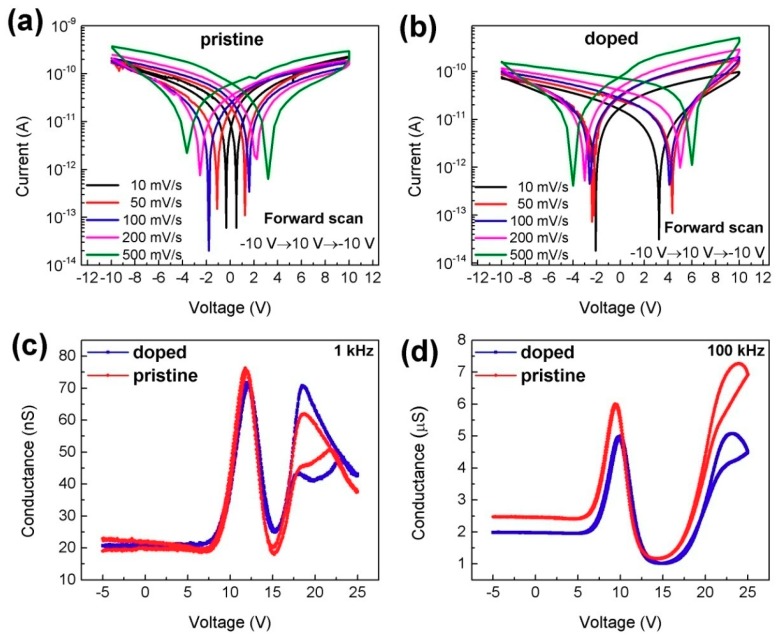
Dark hysteresis curves with various sweep voltage rates and sweep frequencies for iodine-doped perovskite and pristine perovskite based on the vertical structure. (**a**,**b**) Forward dark current voltage hysteresis curves of iodine-doped perovskite and pristine perovskite with various scan rates, respectively. Dark conductance voltage hysteresis curves of perovskite for different sweep frequency; (**c**) 1 kHz; (**d**) 100 kHz.

Insights about time- and frequency-dependent dark hysteresis [12,20] were provided by discriminating the trajectory of dark *I-V* hysteresis response while changing the scan rate and dark conductance-voltage (*G-V*) response with different sweep frequencies. In Figure 4a,b, the *I-V* characteristics show a butterfly-shaped hysteresis loop whenthe voltage is scanned in the sequence −10→10→−10 V (forward scan) at different scan voltage rates. As the voltage sweep rate increases, the zero drift becomes more severe and the saturation current also increases, which can be attributed to both the ion current and the electronic current participating in the conduction mechanism, which is consistent with the vertical structure but different from the lateral structure reported previously [38]. Additionally, *I-V* curves with faster sweep rates showed pronounced hysteresis and larger open-circuit voltages, which are defined by the difference of voltage between the positive drift voltage and negative drift voltage. Iodine-doped perovskite devices exhibited significant dark hysteresis at the same scanning voltage step size compared to pristine perovskite-based devices, as can be observed by comparing Figure 4a,b, which is consistent with the conclusions mentioned above. The direction of the voltage sweep also has a certain influence on the hysteresis [19,20]. The reverse scan (10→−10→10 V) dark hysteresis curves of iodine-doped perovskite and pristine perovskite were employed as shown in Figure S6. There is a slight difference in the current magnitude; namely, the direction of the voltage sweep is basically not related to the zero drift. Ions dominate at low scan rates, consistent with changes in dielectric constant at low frequencies [11,39]. In addition, the current flow in the iodine-doped perovskite is generally lower than that of in the pristine perovskite at the same voltage, which is due to the ion migration that causes the accumulated ions in the perovskite film near the ion-blocking interface to cause a greater degree of p- and n-doping, which partially weakens the applied voltage [12,20,23]. Additionally, samples doped with different iodine contents were also tested, and the results showed that the zero drift amount did not change substantially with the iodine-doped content, which may be due to the maximum solubility of the DMF solution (Figure S7).

Conductance-voltage is often used in molecular electronics to quantitatively measure the conductivity of the junction or the channel [40,41]. Here, *G-V* measurement was employed to describe the ionic conductance. To our knowledge, the *G-V* curve was first used to characterize perovskite materials. Figure 4c,d shows the dark *G-V* hysteresis curves of iodine-doped perovskite and pristine perovskite with sweep frequenciesof 1 kHz and 100 kHz, respectively. Large hysteresis occurs at low frequencies independent of doping, as can be observed by comparing Figure 4c and 4d; this is consistent with the dielectric response characteristics. Additionally, conductance increases with frequency by two orders of magnitude, which reflects the changes in ion conductance at different scan frequencies. More importantly, the hysteresis characteristics become more pronounced after doping the iodine ions, whichis consistent with the *I-V* curves. *G-V* characteristics with various sweep voltage rates at low and high frequency were also executed, as shown in Figure S8. With the increase of scan voltage rates, the hysteresis loops get bigger, which is consistent with the *I-V* curve, but the conductance values decrease.

The apparent hysteresis loop in the *I-V* and *G-V* curves seems to decrease as the scan rate decreases, which is consistent with the reported hysteresis of perovskite solar cells containing electron- and hole-transport layers [20,42]. In addition, the characteristics of the *I-V* properties of the composite structure in the dark are similar to those of complex photovoltaic devices under light, suggesting that the phenomenon origin of the hysteresis properties of photovoltaic devices may be inherent to the perovskite layer. This further suggests that the lag in photovoltaic devices is less likely to be due to ferroelectric behavior [43] or the difference in photocarrier extraction in the electron- or hole-transport layer [44].

To avoid the contingency of this phenomenon, we tested another 10 devices (5 for each perovskite). The *I-V* curves based on the vertical structure with ascan voltage rate of 50 mV/s are shown in Figure S9. As can be seen, the zero drift value of iodine-doped perovskite-based devices is generally larger than the zero drift of pristine perovskite-based devices, which confirms the reliability of this phenomenon. To illustrate this difference more intuitively, statistics of the positive and negative drift voltages and current differences at 0 V of the 10 devices extracted from Figure S9 are plotted in Figure S10. The iodine-doped perovskite-based device not only has a large positive and negative drift voltage, but also has a large current difference at 0 V [19], indicating that it theoretically has a large open-circuit voltage and short-circuit current in iodine-doped perovskite-based solar cells.

The possible origin of sucha large zero drift (hysteresis loop) may be the difference in the defect or vacancy concentration within the perovskite film. The low formation energy of perovskite vacancy defects makes the free surface or grain boundary more susceptible to defects than the bulk, which is particularly important. Recently, Komesu et al. [45] used angle-resolved photoemission spectroscopy and reverse photoelectron emission spectroscopy to find that the surface of the MAPbBr_3_ single crystal has a higher vacancy concentration than the bulk, which leads to an n-type surface appearance rather than the expected p-type characteristics. In another study, Wu et al. [46] also observed higher surface defect densities, two orders of magnitude higher than bulk of MAPbI_3_ and MAPbBr_3_ single crystals. Therefore, this large zero drift originates from vacancy-mediated ion migration and accumulation of ions.

**Figure 5:**
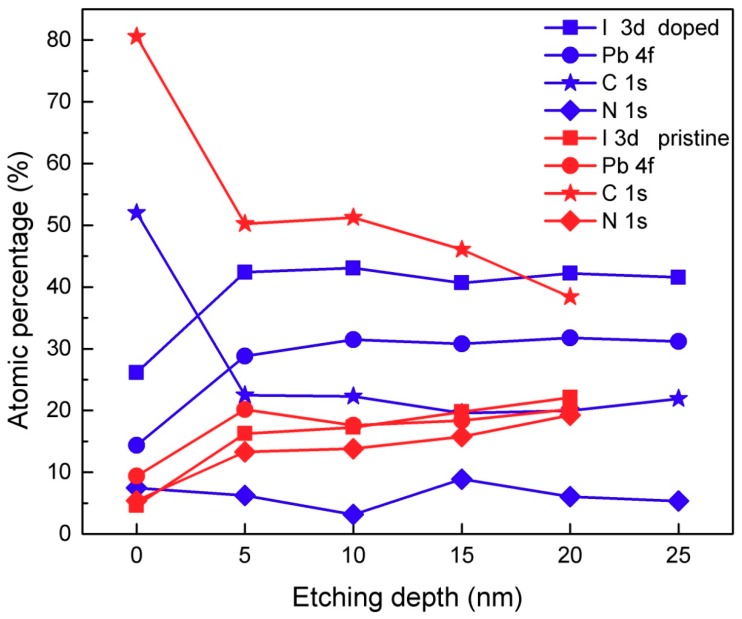
Atomic percentage of iodine-doped perovskite system and pristine perovskite system with the etching depth extracted from the XPS survey spectra.

To directly identify the causes of large zero drift and the effect of iodine ion content on hysteresis, X-ray photoelectron spectroscopy (XPS) was employed to qualitatively analyze the chemical state and to analyze the elemental distributions in the iodine-doped perovskite and pristine perovskite. The data was calibrated using the electronic binding energy of 284.6 eV of C 1s, and the binding energy profile was smoothed using the Wiener Filter mode. The survey spectrum and FWHM of the iodine-doped perovskite and pristine perovskite with etching depth are shown in Figure S11 and Table S1, respectively. The corresponding atomic contents extracted from the survey spectra are shown in Figure 5. The surfaces of the pristine and doped perovskite clearly deviate from the bulk with regard to the observed C and I content. The other elements remained basically unchanged. The C content decreases drastically to a stable value during etching, indicating significant surface contamination remaining from the fabrication process. The I content of the doped perovskite is always higher than that in the pristine. For a more intuitive explanation of ion (vacancy) concentration gradients, the I/Pb ratio was extracted from the XPS survey spectrum listed in Table 1. With the increase of etching depth, the I/Pb ratio in iodine-doped perovskite decreases, while it increases for pristine perovskite. This indicates that the accumulation and depletion of iodine mainly accumulates on the surface, rather than the bulk, of perovskite films. Ion migration under an electric field requires the aid of defects. A large number of vacancies without ions do not cause large hysteresis. Similarly, a large number of ions without vacancies do not cause large hysteresis. High concentrations of vacancies and high concentrations of ions coexisting at the same time cause large hysteresis [10,21]. The ideal chemical composition with an average atomic ratio between iodine and lead is 3:1; however, the pristine perovskite is iodine poor due to the low activation energy of iodine. In the process of iodine doping, the following reaction occurs in the precursor solution [47]:

(1) $$I^{-}+I_{2}\;{}_{\leftarrow }^{\to }\;I_{3}^{-}$$

This will cause the iodine-lead atomic ratio to be much lower than the ideal value. For the doped perovskite, the iodine-lead atomic ratio is greater than that of pristine perovskite, leading to the large zero drift and suggesting that iodine ions are the culprit causing current hysteresis.

**Figure 6:**
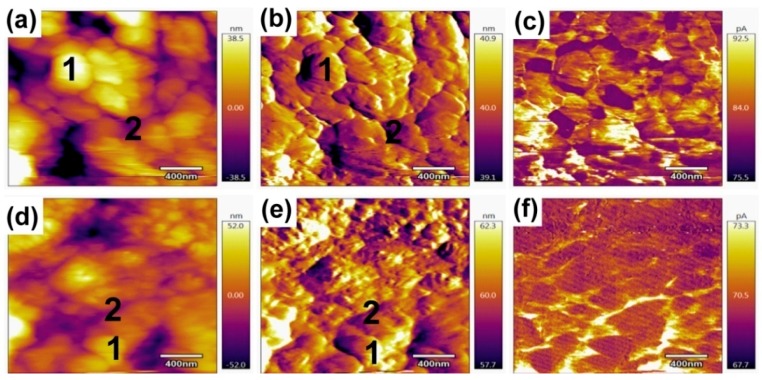
Grain boundary morphology dependent current measured by c-AFM for iodine-doped perovskite films (**a**–**c**) and pristine perovskite films (**d**–**f**). (**a**) Height and (**b**) deflection maps of the same area of iodine-doped perovskite; (**c**) Dark current measured with the c-AFM tip in contacting with the iodine-doped perovskite; (**d**) Height and (**e**) deflection maps of the same area of pristine perovskite; (**f**) Dark current measured with the c-AFM tip in contacting with the pristine perovskite.

Grain boundaries can be dominant pathways for ion migration in perovskite films, which might lead to differences in the measured values of activation energies [22,29,33,48]. To detect the ion transport at the grain boundaries of iodine-doped perovskite and pristine perovskite, local ion currents related to ion migration on grain boundary and particles were measured using a conductive atomic force microscope (c-AFM) under dark conditions. The topography of the iodine-doped perovskite and pristine perovskite films are shown in Figure 6a,b,d,e. It can be observed that there are two different types of grains: one showing a smooth surface (#1) and the other showing a layered texture (#2). Iodine-doped perovskite shows a better surface flatness than pristine perovskite, which is in accordance with the results of SEM. The dark current of the bare perovskite film was measured with ascanning tip bias of 3 V (Figure 6c,f). The currents measured at #1 and #2 are called “grain internal current” and “grain boundary current”, respectively. The grain boundary current has the same order of magnitude as the current difference at 0 V, indicating that grain boundary current is dominated by ion migration current in the dark. It is noted that there is variation of dark current at different grain boundaries between the iodine-doped perovskite and pristine perovskite, suggesting the iodine-doped perovskite has a large current hysteresis due to its large ion current, which also explains the giant zero-point drift voltage in iodine-doped perovskite. 

## 4. Conclusions

In summary, we demonstrated the effect of doping properties on the hysteresis loop and zero drift by doping the native species (iodine ions) onto the CH_3_NH_3_PbI_3_ perovskite. Iodine-doped perovskites exhibit large zero-point drift in both vertical and lateral structures through current-voltage under dark conditions. Sweep-dependent hysteresis characteristics indicate that dark hysteresis and zero drift increases as the scan rate increases; more importantly, ion migration dominates at low scan rates, as determined by *I-V* characteristics, and low sweep frequency, as determined by *G-V* characteristics. XPS results show that the content of iodine and lead in iodine-doped perovskite remains basically unchanged with an increase of etching depth, while the content of iodine and lead in pristine perovskite increases with the increase of etching depth. The iodine-lead atomic ratio decreases with increasing etch depth in the iodine-doped perovskite, whereas for the pristine perovskite it is the opposite. The amount of iodine ions in iodine-doped perovskites is greater than that of the pristine perovskite, making the iodine-doped perovskite produce large ion hysteresis, which explains the large zero drift in iodine-doped perovskites. Conductive atomic force microscopy was used to measure the perovskite grain boundaries and grain interior currents, indicating that the iodine-doped perovskite has a larger ion current than the pristine perovskite, further confirming the larger zero drift in the iodine-doped perovskite. Most of the previous reports on resistive switching devices use inorganic oxides such as SiO_2_ [49], TiO_2_ [50,51] and Ga_2_O_3_ [52,53] as the resistance-switching layers. Our results prove that the iodine-lead ratio can be artificially adjusted to obtain large ionic conductivity so that the iodine-doped perovskite can be used as a candidate for memristors and solid electrolytes [54] in ion batteries.

## Figures and Tables

**Table 1:** I/Pb atomic ratio of iodine-doped perovskite and pristine perovskite with the etching depth.

I/Pb Atomic Ratio	0 nm	5 nm	10 nm	15 nm	20 nm	25 nm
Iodine-doped perovskite	1.812	1.428	1.324	1.312	1.300	1.285
Pristine perovskite	0.494	0.806	0.981	1.077	1.092	-

## Supplementary Materials

The following are available online at http://www.mdpi.com/1996-1944/11/9/1606/s1.
