# Supplementary material for: Giant Zero-Drift Electronic Behaviors in Methylammonium Lead Halide Perovskite Diodes by Doping Iodine Ions
Source: Materials (Basel). 2018 Sep 4;11(9):1606. doi: 10.3390/ma11091606 (PMC6163366; doi:10.3390/ma11091606)
Supplement: Supplementary file 1 [file materials-11-01606-s001.zip › materials-346696-SI.pdf]

materials

Supporting Materials:

Giant Zero-Drift Electronic Behaviors in Methylammonium Lead Halide Perovskite Diodes by Doping Iodine Ions

Tiqiang Pang [1], Renxu Jia [1,*], Yucheng Wang [1], Kai Sun [2], Ziyang Hu [2,*], Yuejin Zhu [2], Suzhen Luan [1] and Yuming Zhang [1]

1 School of Microelectronics, Xidian University, Key Laboratory of Wide Band-Gap Semiconductor Materials and Devices, Xi'an 710071, China; tqpang@stu.xidian.edu.cn (T.P.); ycwang_23@stu.xidian.edu.cn (Y.W.); luansuzhen@126.com (S.L.); zhangym@xidian.edu.cn (Y.Z.)
2 Department of Microelectronic Science and Engineering, Ningbo University, Ningbo 315211, China; sky1250565776@sina.com (K.S.); zhuyuejin@nbu.edu.cn (Y.Z.)
* Correspondence: rxjia@mail.xidian.edu.cn (R.J.); huziyang@nbu.edu.cn (Z.H.)

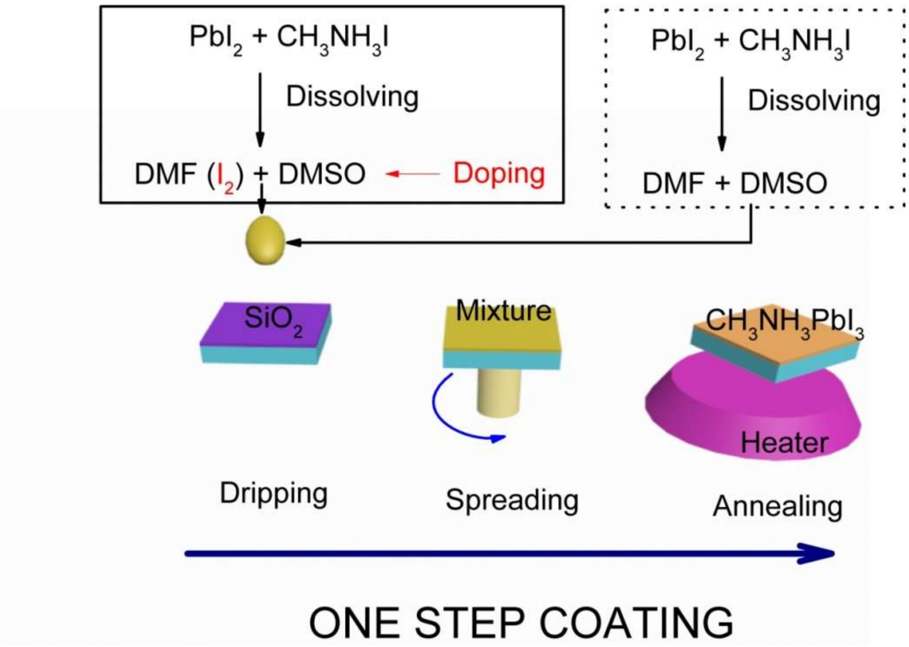

Figure S1. One-step coating procedure to deposit $CH_3NH_3PbI_3$ perovskite and iodine doped perovskite films.

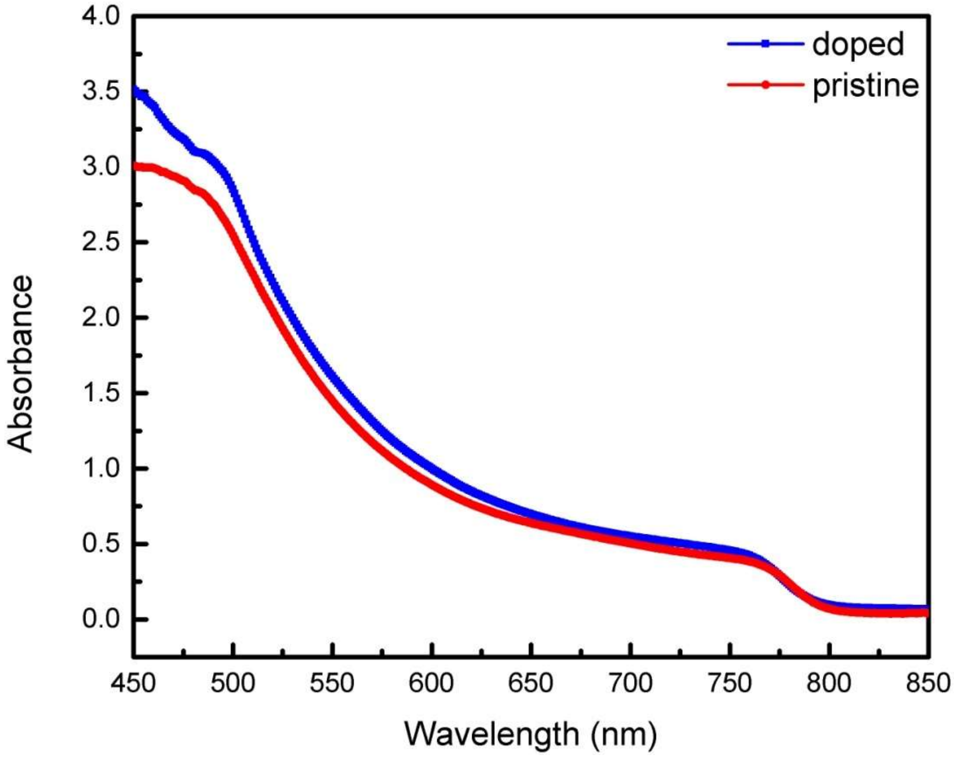

Figure S2. UV-visible absorption spectra of 300 nm perovskite thin film on transparent substrates. The red stand for the pristine perovskite film and the black representative the iodine doped perovskite film.

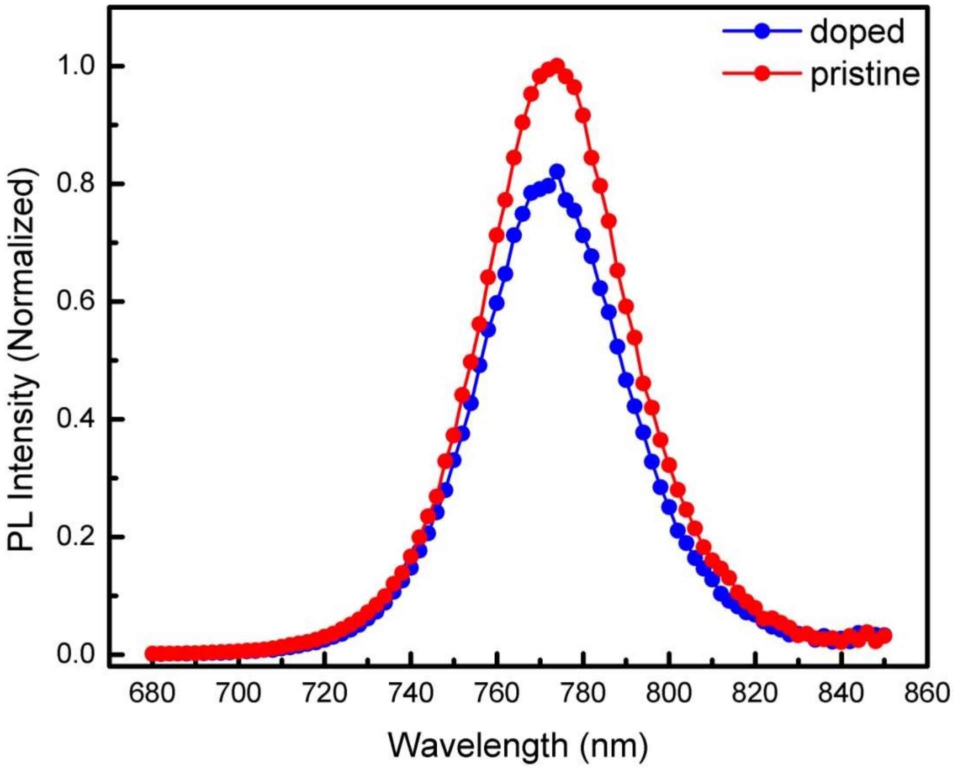

Figure S3. Steady state photoluminescence spectrum of the as-grown hybrid perovskite film. The higher fluorescence efficiency is pristine perovskite, the other is iodine doped perovskite.

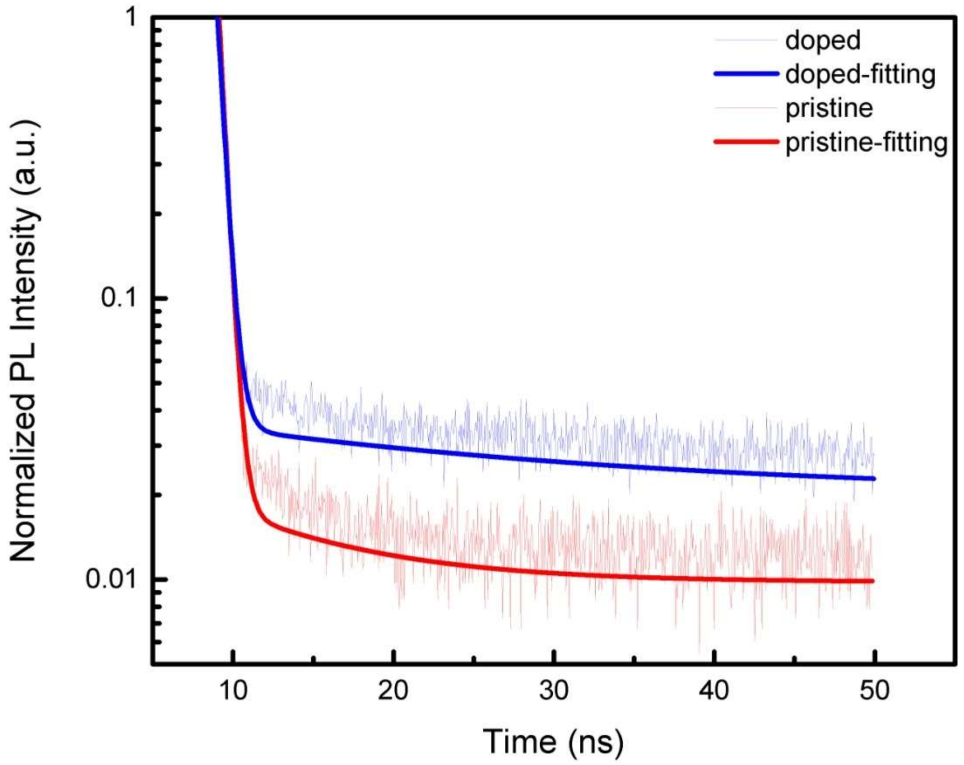

Figure S4. Transient fluorescence spectroscopy of pristine perovskite film (the blue) and iodine doped perovskite film (the red). The thick solid line in blue and in red are the fitting curves of the pristine perovskite film and the iodine doped perovskite film, respectively, with the method of biexponential decay fit.

UV-visible absorption spectra of 300 nm perovskite thin films on *fluorine doped tin oxide (FTO) substrates* were shown in **Figure S2**. In spite of the same band edge absorption, the absorption characteristics of iodine doped perovskite are clearly better than that of pristine perovskite in the short wavelength range. Steady-state fluorescence spectroscopy of perovskite films were compared to investigate the impact of iodine ion content on carrier depopulation as shown in **Figure S3**. It is evidenced that the iodine doped perovskite fluorescence emission peak (774 nm) *excited with the wavelength of 465 nm* is the same with that of the pristine perovskite film, demonstrating that the bandgap of perovskite is not influenced by the iodine ion content which is agreement with the results of absorption spectroscopy. The fluorescence intensity implies that pristine perovskite has stronger recombination efficiency than iodine doped perovskite due to the assistance of vacancy defects. The time-resolved photoluminescence decay of the pristine perovskite and iodine doped perovskite were shown in **Figure S4**, measuring the peak emission at ~774 nm fitting by biexponential decay. The inset is the fitting time parameters. The photoluminescence decay of the iodine doped perovskite film exhibits a time constant (τ) 24 ± 5 ns. In contrast, the lifetime for the pristine perovskite film is only 8 ± 1 ns. The iodine doped perovskite had large charge transient time than the pristine perovskite, it can be attributed to the decrease of the trap state density caused by the *reduce* of iodine vacancy.

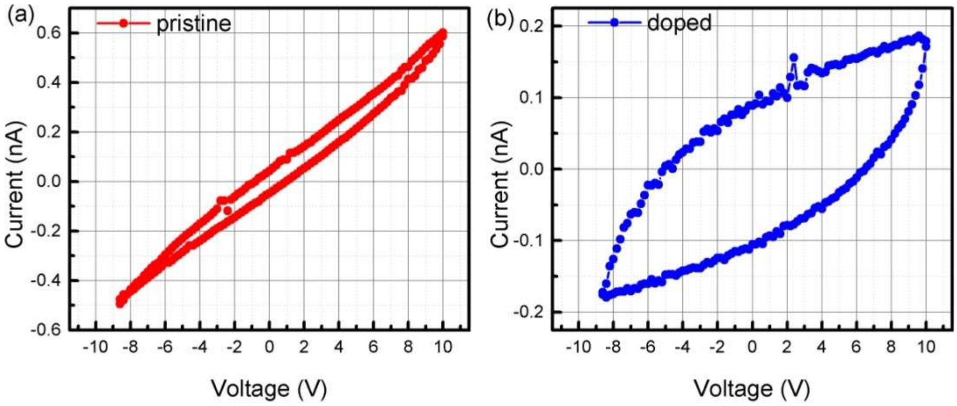

Figure S5. *I-V* hysteresis curves of (a) without doping perovskite and (b) iodine doped perovskite based on the vertical structure in linear coordinates.

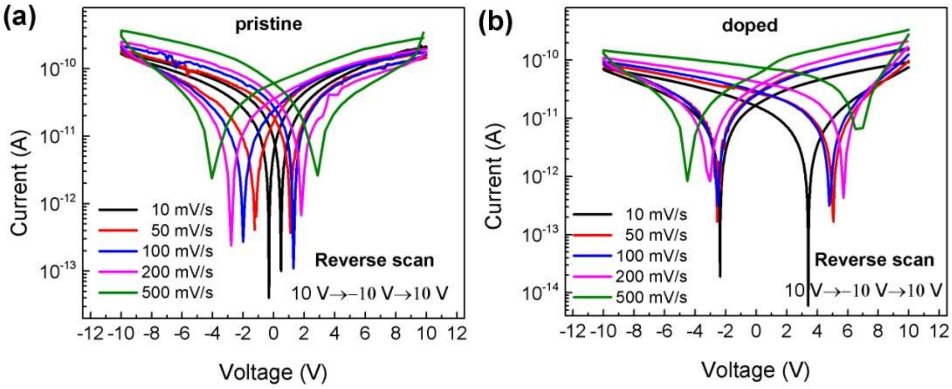

Figure S6. Reserve dark *I-V* hysteresis curves with various sweep voltage rates for (a) pristine perovskite and (b) iodine doped perovskite based on the vertical structure, respectively.

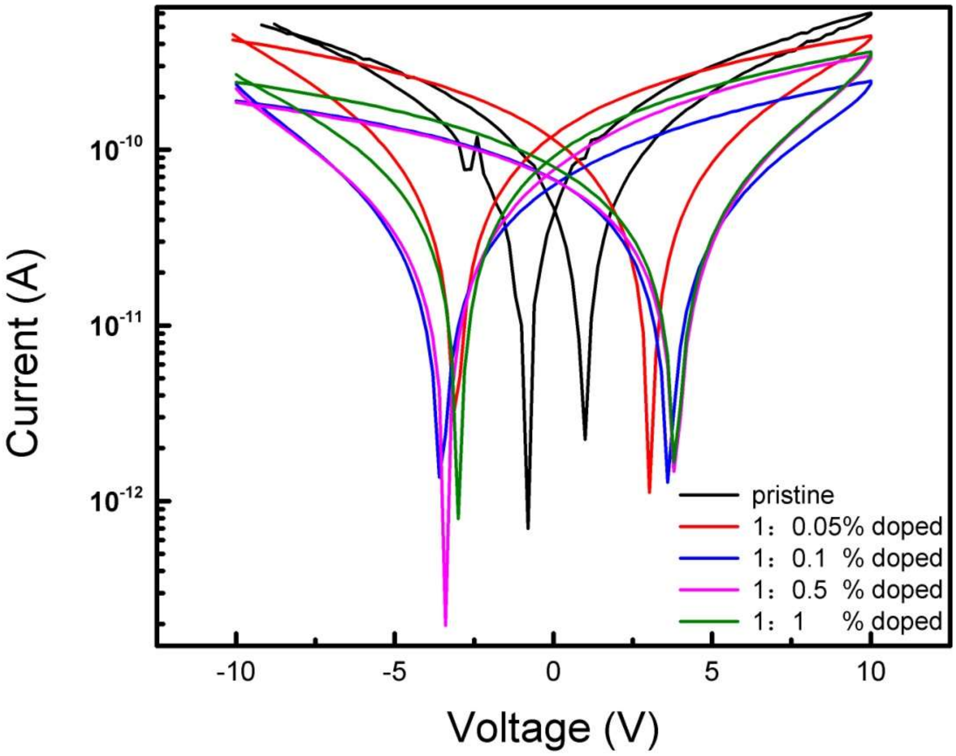

Figure S7. *I-V* hysteresis curves of doped perovskite with various iodine content based on the vertical structure.

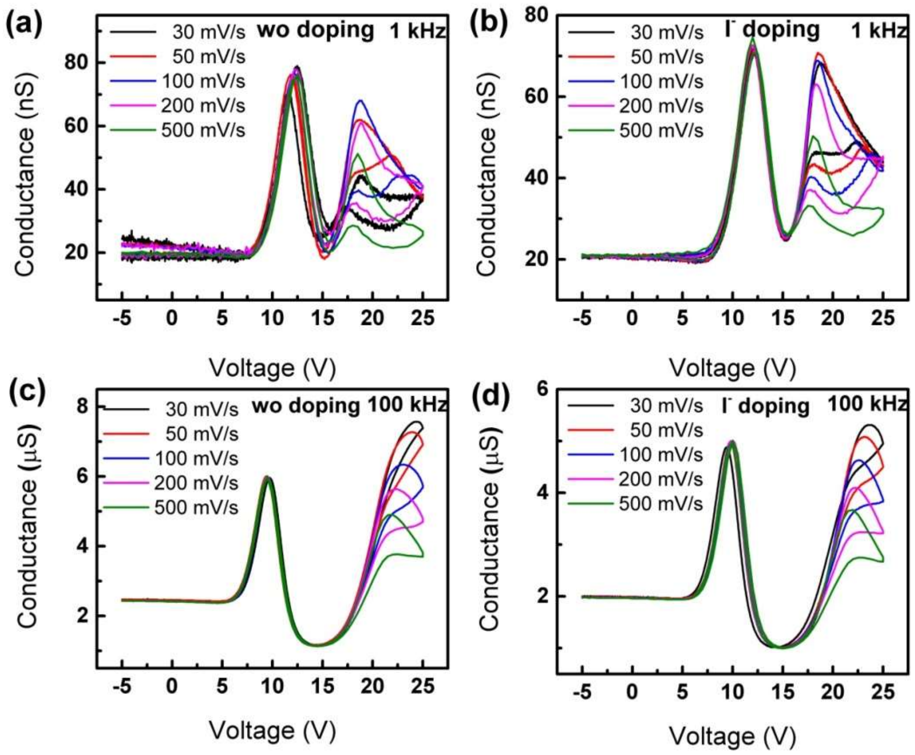

Figure S8. Dark conductance voltage hysteresis curves with various sweep voltage rates based on the vertical structure. (a) pristine perovskite at 1 kHz. (b) iodine doped perovskite at 1 kHz. (c) pristine perovskite at 100 kHz. (d) iodine doped perovskite at 100 kHz.

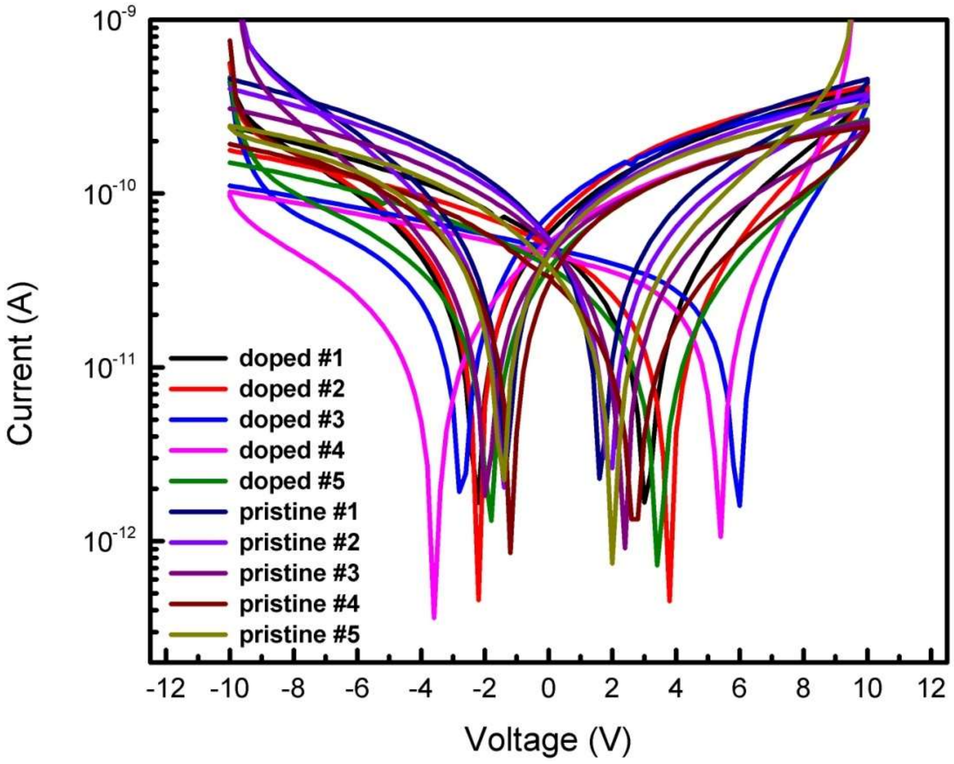

Figure S9. Reliability and statistics of hysteresis caused by voltage drift. (a) Dark *I-V* hysteresis curves of iodine doped perovskite and pristine perovskite based on the vertical structure for each another five devices.

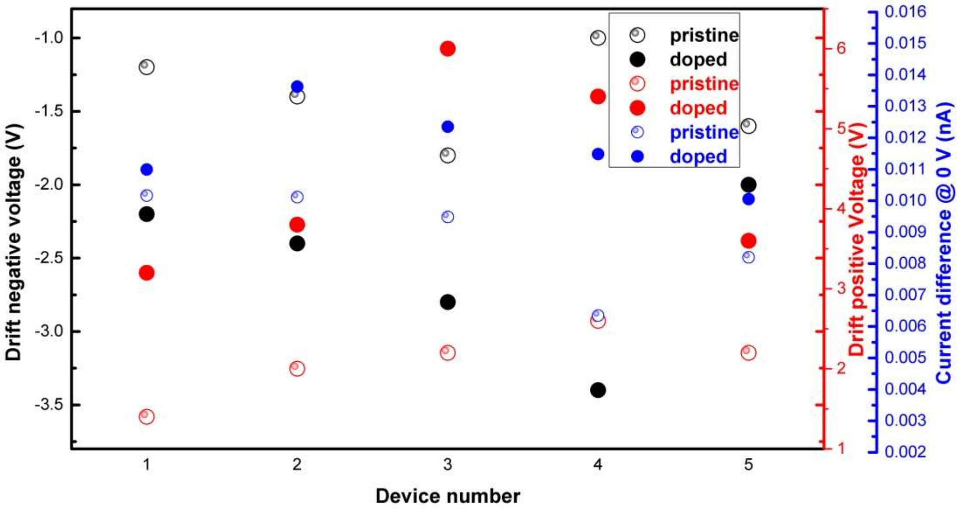

Figure S10. Statistics of drift positive voltage, drift negative voltage and current difference at 0 V extract from Figure S9.

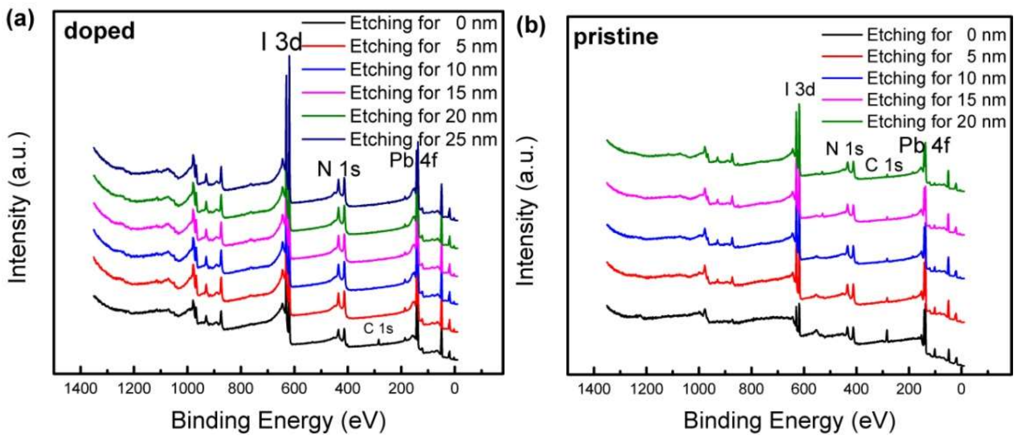

Figure S11. XPS spectrum of the iodine doped perovskite and pristine perovskite with etching depth. (a) XPS survey spectrum of iodine doped perovskite with the 25 nm etching depth. (b) XPS survey spectrum of pristine perovskite with the 20 nm etching depth.

Table S1. FWHM of iodine doped perovskite and pristine perovskite with the etching depth.

Type	Element	0 nm	5 nm	10 nm	15 nm	20 nm	25 nm
**Iodine doped perovskite**	**I**	**2.53 *eV***	**2.54 *eV***	**2.54 *eV***	**2.54 *eV***	**2.55 *eV***	**2.54 *eV***
	**Pb**	**2.55 *eV***	**2.84 *eV***	**2.97 *eV***	**3.06 *eV***	**3.07 *eV***	**3.12 *eV***
	**C**	**3.05 *eV***	**3.40 *eV***	**10.86 *eV***	**3.10 *eV***	**2.08 *eV***	**4.13 *eV***
	**N**	**1.47 *eV***	**2.50 *eV***	**2.34 *eV***	**3.58 *eV***	**1.00 *eV***	**3.58 *eV***
**Pristine perovskite**	**I**	**2.81 *eV***	**2.92 *eV***	**2.68 *eV***	**1.51 *eV***	**2.58 *eV***	**-**
	**Pb**	**1.59 *eV***	**2.48 *eV***	**2.79 *eV***	**1.93 *eV***	**3.11 *eV***	**-**
	**C**	**3.50 *eV***	**3.74 *eV***	**3.13 *eV***	**3.08 *eV***	**3.48 *eV***	**-**
	**N**	**1.59 *eV***	**0.93 *eV***	**1.00 *eV***	**0.98 *eV***	**1.27 *eV***	**-**
